# Report of the Committee of the Pennsylvania Society of Dental Sugeons, on Dr. Evans Amalgam

**Published:** 1850-07

**Authors:** 


From the Dental News Letter.

### REPORT OF THE COMMITTEE OF THE PENNSYLVANIA SOCIETY OF DENTAL SUGEONS,

### ON DR. EVANS AMALGAM.

*To the President and Members of the Pennsylvania Association of Surgeon Dentists:*

Gentlemen—Your committee, appointed at the stated meeting, held on the 6th of February, 1849, and to which was referred Dr. T. W. Evans' Amalgam, respectfully beg leave to report, that they have attended to that duty, and submit the following conclusions, to which they have arrived, viz:

1st. As to the general question of the use of amalgam, and other compounds of the baser metals and pastes, as a filling for teeth, your committee would not wish to be comprehended as restricting any member of the profession in their occasional use, as directed by the exercise of their deliberate judgment; but as a general substance for filling, would condemn it, and would in any case recommend the observance of great caution. Notwithstanding the violent and much to be deplored dissensions which have existed for the last few years among the most eminent in the whole domain of the protession, still, the subject of these preparations for fillings has received the closest scrutiny, and the most extensive and multiplied experiment, and sufficiently careful to have settled beyond the question of controversy, the impropriety of uniting any two kinds of metals whatever in the same filling, or the same tooth. Those dissensions have had their beneficial influence, however, in a two-fold light—first, that of directing the most careful inquiry into a subject of the greatest magnitude to the profession; and secondly, by the publication, through the journals of the country, of the separate views of the different observers, have also contributed to enlighten the reading public. And as far as your committee are able to judge, the most intelligent class of the community receive with suspicion any compounded material for filling teeth, and mostly refuse, positively, to submit to its employment.

Although your committee were appointed to consider the subject of Evans' Amalgam only, still, as it is so identical with most others that have been, and are still in use, except that it contains *cadmium*, they consider it a fitting occasion to remark upon amalgams and compounds as a general subject, and express their convictions, with a view of settling the matter, as far as those substances are concerned, at once; and moreover, as the most objectionable and important ingredient in it is also contained in most others, viz.: *mercury*. Now, as to the deleterious influence of mercury on some constitutions, there remains no longer a doubt, even though received into the system by the slow process of the decomposition of a plug or a number of plugs in the teeth of a patient. In fact, cases are constantly coming to light, through the most respectable sources, sustaining this conclusion, and as Evans' Amalgam, as well as all others, are destroyed by the action of the acidulated secretions of the mouth, in some or most patients, it loses its value as a safe material for filling teeth. Yet your committee are aware that some patients do not present the appearance of suffering from its use; still the operator cannot know, by any external or other signs, in which or what kind of constitution or temperament, it may with safety be employed.

2dly. With regard to the merits of Evans' Amalgam. Your committee have been anticipated by the author, in a communication to the proprietors of the "Dental News Letter," under date, Paris, Dec. 11, 1849, in which he holds the following language:—"Finding it to differ so much in different cases, I am induced to regard it as at best an uncertain article. I do not feel satisfied to use it, even as an expedient, under such circumstances; having no confidence myself in its durability, I do not feel justified in recommending its use to the profession." Notwithstanding the renouncement by the author, he makes use of the following commendatory language in a communication to the proprietors of the journal named above, under date, London, April 20, 1849: "The first in the profession in London have pronounced it the very best ever invented. Finding this, I cannot feel myself justified in withholding it from the profession. I propose publishing it freely. I have never had anything belonging to dental science that I wished to conceal, and this being an article intended to benefit humanity, I therefore wish every one to be the possessor of it. I think it must supplant the many substances which are used, most of which I cannot but feel are very deleterious; this, I *know* is not." He further adds a series of reasons why it is superior to any heretofore in use, and claims for it a number of special merits, among which the subjoined are the most important. 1st. "There is in it no ingredient that can possibly render it improper to be employed in the most delicate constitution; it is perfectly harmless, both as it respects the general health and the teeth themselves." 2dly. "Almost *immediately* after introduction into the cavity, it becomes hard, and as it hardens it *expands*." 3dly. "A cavity filled with this compound is altogether impermeable to the fluids of the mouth, and strong tests have proved its perfect insolubility," and 4thly, "The most delicate comparison of the weight of the filling at the time of insertion, with its weight after having been in the mouth, proves that it undergoes no change whatever in this respect."

Now, with regard to the first and important merit claimed for it, your committee would dissent in the strongest terms, and it is the first time your committee have heard any one claiming that mercury is not "improper to be employed in the most delicate constitutions, as respects the general health or the teeth themselves."

The citation of the following case will fully illustrate many of its characteristics as a useless substance, and explain nearly all points of objection to it: A lady of twenty-five years of age, high nervo-sanguine temperament, in general good health, teeth much disposed to decay, highly sensitive, and would not hold plugs well, had the second right superior molar plugged on the back part and palatine surface—the plug supplying about two-thirds of the whole substance of the tooth; the nerve was dead in the tooth, and it had been plugged for some time with tin or gold, and answered the purpose of attachment of a partial set of artificial teeth for a long time; it never had been sore in the gum. It was filled with great care, and was understood by the patient as an experiment with a new and highly extolled amalgam. Upon putting the tongue to it there was a very strange sensation—a peculiar, pungent and cold sensation—which was very much increased on putting the band on the tooth. This became a source of considerable annoyance for some weeks, when it diminished by degrees, leaving the tooth sore in the gum, somewhat loose, and with pain and uneasiness all the time; finally, in about three months, the plug commenced crumbling out, and the first sensations passed off—that of cold pungency. The balance of the plug was removed, and presented the appearance of gray ashes, the mercury had been entirely absorbed. There is not the least doubt but that the secretions of the mouth operated to dissolve this plug very rapidly. The adjacent teeth, as well as the plugged one, were quite yellow. The same tooth was plugged with tin in a few days, with a great deal of labor, when none of these symptoms were experienced, the sponginess of the gums left, and the tooth is firm. Three cases out of four terminated in this way.* On the other hand, a gentleman fifty years of age, of general good health and good constitution, had the back part of the second inferior molar (nerve dead) plugged, and it comes in partial contact with the food in chewing, does not produce any unpleasant symptoms, but is not as good as gold or tin in the same place, as it wears away very fast. They have all complained of a cold sensation about the teeth plugged with it, Now, under such circumstances, all its "peculiar merits," as to "impermeability," its harmlessness in the most "delicate constitutions," maintaining its specific gravity, etc., etc., fall, with the plugs, *to pieces*. And all that is left for the committee to say is, to compliment the generous and liberal feeling which stimulate the inventor to lay it freely before the public. If all

* A young lady, twenty-five years of age, general good health, nervo-bilious temperament, teeth generally good, and hold plugs well. Inferior left second molar, large plug, nerve not destroyed, been plugged about one year, has never given any unpleasant symptoms, does not wear any, but is not used in masticating; gives satisfaction.

were to appreciate the lesson this substance and the inventor's course affords them, there would be many more good things in use, and many more bad ones out of use.

All of which is respectfully submitted.

*Resolved*, That while your committee do not wish to restrict any member of the profession in the use of amalgam, as a temporary filling, knowing that a discerning public will govern him in that respect, as regards its use, to the exclusion of gold —more than all the laws and resolutions a society could adopt; still, they would recommend the entire abandonment of it as a safe and permanent filling for teeth.

F. REINSTEIN,<br>

C. C. WILLIAMS,<br>

W. R. WHITE,<br>

S. L. MINTZER,<br>

A. R. JOHNSON, } Committee.

